# Ubuntu in Namibia and Kenya: How Emerging Adults Live an Essential African Value Today

**DOI:** 10.1177/00220221241309863

**Published:** 2025-01-20

**Authors:** Julia S. Rotzinger, Lene Arnett Jensen, Amber Gayle Thalmayer

**Affiliations:** 1 University of Zurich, Switzerland; 2 Clark University, Worcester, MA, USA

**Keywords:** cultural psychology, sub-Saharan Africa, Ubuntu, emerging adulthood, Namibia, Kenya, African psychology, values, African communalism, cultural-developmental approach

## Abstract

Psychology in Africa often relies on Euro-American theories, despite their limitations in African cultures. Here, a shift to an Afrocentric perspective was made by exploring *Ubuntu* in two sub-Saharan African countries using a cultural-developmental psychology approach for in-depth emic understanding. Ubuntu (also known as *botho*, etc.) is a moral ideal that at core means placing a higher importance on the community than on oneself. Most literature on this important African concept has been theoretical rather than empirical, and little is known about how well it describes contemporary worldviews and experiences of emerging adults. In this interview study, Namibian and Kenyan emerging adults (ages 18–20 years, *N* = 23) shared their views on, identification with, and behaviors shaped by Ubuntu values. Reflexive thematic analysis was used to develop four themes that convey the advantages and disadvantages described: I am because I am connected; Together we do better; Following and passing on traditions; and Costs of community—freeloaders and restrictions. Findings support the theoretical literature on Ubuntu while contributing the voices of a new generation, coming of age into a mix of tradition and globalization. Participants identified strongly with Ubuntu values and practiced them in old and new ways. They also sometimes desired more autonomy in the realms of education and spirituality. This empirical study on Ubuntu offers insights into the lived experience of a philosophy and values of ongoing importance in the African region and sets the stage for a research agenda to integrate this important construct into cross-cultural psychology.

Only a tiny percentage of research published in major psychology journals includes African participants or authors (Thalmayer et al., 2021). Consequently, it is not surprising that psychological research and academic training in Africa also largely rely on European and American theories and findings, despite their limitations in African contexts (Nwoye, 2015). There is a profound need to understand indigenous African concepts, especially given Africa’s sizable and growing population (International Monetary Fund, 2023). There is also no reason African concepts should not be a source of theory for global psychology (Adetula et al., 2022). As one prominent African scholar observed, “Africa deserves and ought to be an integral part of evolving an inclusive science of psychology” (Nsamenang, 2015).

The present research uses a cultural-developmental psychology approach to gain an in-depth emic (localized, bottom-up) understanding of *Ubuntu* among emerging adults in Namibia and Kenya (Jensen, 2011a, 2020a). Ubuntu, known by a variety of different translations, as described below, is a deeply meaningful concept across many African cultures. Nelson Mandela (2017) embraced it when he stated: “In Africa there is a concept known as Ubuntu, based upon the recognition that we are only people because of other people.” Yet, this concept has received almost no empirical attention in the field of psychology. The mainly theoretical treatments of the concept come from philosophy, law, and education (Sodi et al., 2021). Exploring Ubuntu in the lived experience and psychology of emerging adults in African contexts in this study is intended as a shift toward the inclusion of Afrocentric perspectives in empirical psychological research (Adetula et al., 2022; Ratele, 2017; Serpell et al., 2022).

Emerging adulthood, the period from approximately 18–29 years of age, is a developmental period where identities often are explored and formed (Arnett, 2000, 2024). Research on emerging adulthood in Africa is only recently beginning to appear (e.g., Lo-oh, 2016; Obidoa et al., 2019; Theron et al., 2021; Willmore et al., 2023). Here, the focus on emerging adults provides an opportunity to examine if and how the philosophy of Ubuntu is internalized or questioned as part of identity development.

## An African Concept Across Countries and Social Contexts

Sub-Saharan Africa has great cultural diversity, exemplified by its languages: around 2,000 are currently spoken, representing nearly one third of the world’s living languages (Brown & Ogilvie, 2010). Despite this diversity, numerous pan-African cultural similarities exist, for example, among the predominant Bantu-language groups (Brown & Ogilvie, 2010; Fortes-Lima et al., 2024). Pan-African cultural similarity can also be seen in Ubuntu philosophy and values (e.g., Adjei, 2019). Although the term Ubuntu comes from the Xhosa/Zulu culture of South Africa, values and behaviors related to it are found across African countries (Mugumbate & Chereni, 2020; Oppenheim, 2012).

African scholars describe Ubuntu as involving humanity, compassion, generosity, love, respect, and collaboration (Mnyaka & Motlhabi, 2005; Sodi et al., 2021). They also agree that Ubuntu encapsulates the “very essence of what it is to be a morally and ethically celebrated African person” (Sodi et al., 2021, p. 442). Ubuntu is a moral ideal that involves feeling and acting humanely toward others, including reciprocity and justice; ultimately, placing a higher importance on the community than on oneself (Dolamo, 2013; Mnyaka & Motlhabi, 2005; Ngondo & Klyueva, 2022; Ukpokolo, 2017; Venter, 2004; Wilson & Williams, 2013). The classic expression, “I am because we are, and ‘since’ we are, therefore I am” (Mugumbate et al., 2023, p. 3) stands in contrast to the tradition of Western individualism exemplified by Descartes’ dictum “I think therefore I am” (Ngondo & Klyueva, 2022, p. 27; see also Mangena, 2016).

Scholars have most typically used the term Ubuntu, but other local terms include *botho* in Sotho language in South Africa, *omundu* in Herero in Namibia, and *utu* in Swahili or *munto* in Meru in Kenya (Mugumbate & Chereni, 2020; Sodi et al., 2021; “Ubuntu Philosophy,” 2024). In Kenya, though not Namibia, Ubuntu is a well-known word. In both countries, *harambee* is a familiar term used in political contexts (described further below) with usage highly synonymous with Ubuntu, though some sources describe harambee as a narrower value pertaining to familyhood and blood relationships (Africa Social Work and Development Network, n.d.).

Ubuntu values are important across social contexts (Bronfenbrenner, 2005). Starting in childhood, families and communities transmit these values through stories, songs, rituals, and education, for example in maxims taught early to children, such as “When webs of a spider join, they can trap a lion” and “Fifty lemons are a load carried by ‘one person,’ but for fifty people they are perfume,” to impart that mutual support and cohesion reduce burdens and solve problems (Mugumbate et al., 2023, p. 4; see also Mupedziswa et al., 2019). They also apply to the communal process of raising and educating children, exemplified in the maxim: “A child belongs to the whole village” (Mugumbate et al., 2023, p. 3; see also Leonard et al., 2022).

The importance of Ubuntu philosophy extends beyond the family and community to include macro-level economic and political contexts, where it plays an important role in organizational management and national policies (Chieni, 1998; Goldman et al., 2019; Nanyeni, 2016). For example, in Namibia, the government introduced the Harambee Prosperity Plan (HPP) in 2016, a political effort aimed at improving the economy, infrastructure, and education by means of teamwork. HPP encourages the Namibian population to work together to achieve common goals (Nanyeni, 2016). In Kenya, harambee has been an important political concept since independence in 1963, encouraging community members to create communal goods and work together on local projects (e.g., infrastructure, schools, health facilities, agriculture; Chieni, 1998; Ngau, 1987). The core values and related practices, however, existed long before: Almost every community in Kenya already had collective groups to facilitate mutual support (Chieni, 1998).

## Ubuntu: Theoretical Perspectives on Key Components

Almost all literature on Ubuntu is theoretical rather than empirical. This literature comes from the fields of philosophy (e.g., More, 2006; Ukpokolo, 2017), psychology (e.g., Mkhize, 2004), law (e.g., Cornell & Muvangua, 2012), and education (e.g., Waghid & Smeyers, 2011). Sodi and colleagues (2021) made an important first step toward the psychological study of Ubuntu (they center the term botho from Sotho languages) by synthesizing the literature and proposing that it comprises four psychological “elements”: African spirituality, personhood, interconnectedness, and communalism. African spirituality, the first element, implies that a person’s spirit is connected to material and immaterial entities, such as humans, ancestors, animals, plants, and God. Personhood means that a person is a person through other people, through relations and connections with other community members, and through communal participation. Interconnectedness highlights the importance of relationships that exist not only between people but also with ancestors, spiritual beings, and the natural environment, while communalism emphasizes the family and community.

Although much of the literature focuses on the positive side of Ubuntu, some authors have noted potential disadvantages (e.g., Van Dyk & Nefale, 2005). Van Dyk and Nefale (2005), for example, suggested that emerging adults who have exposure to a Western lifestyle may experience Ubuntu as pressure to conform to communal expectations rather than following their own goals and interests. As far as we are aware, however, the literature on disadvantages is also solely theoretical rather than empirical. Sodi and colleagues’ (2021) four elements provide a useful framework for approaching the empirical study of this important but broad construct, facilitating a systematic approach to assessing the views on, identification with, and behaviors shaped by Ubuntu values for emerging adults today, including both advantages and disadvantages of this pervasive cultural value.

## Ubuntu Values and Philosophy Among Emerging Adults

Little is known about the extent to which Ubuntu is embraced by the current generation of emerging adults in Africa. Unlike previous generations, contemporary emerging adults have come of age in a globalized world (Jensen, 2011b; McKenzie, 2019; Willmore et al., 2023). Even emerging adults who grew up in traditional rural communities are exposed to Western culture and globalization through media and technology (Gastineau & Golaz, 2016; Jensen, 2022).

In Kenya, emerging adults are sometimes referred to as the “dotcom” or “digital” generation, and they are seen as having new educational and employment opportunities, and to be shifting from traditional to modern ways of life (Moore & Smith, 2020). In Namibia, emerging adults are part of the “born-free,” post-apartheid generation born after independence in 1990. They have more liberties and opportunities than their parents and grandparents did (Smith et al., 2020). Some Namibian emerging adults have been reported to feel a potent responsibility to bring about societal change, while others show limited interest in political and public activities (Lindeke, 2014). Contemporary Kenyan and Namibian emerging adults constitute a generation whose experiences are very likely more diverse than those of previous generations and who must balance the values and traditions of their elders with the pressure and opportunities of a globalizing world.

Using a cultural-developmental psychology approach, interviews were conducted with emerging adults in two countries, Namibia, and Kenya, to gain in-depth, emic understanding of the views on, identification with, and behaviors shaped by Ubuntu values. Although the experience of emerging adulthood varies across cultures, it is often a period of identity exploration and formation, including with regard to values (Arnett, 2000, 2024; Lo-oh, 2016; Obidoa et al., 2019). Thus, emerging adults are of particular interest because they can provide insight into the extent of continuity and changes to Ubuntu values in Namibia and Kenya. We considered it important to conduct a qualitative interview study at this stage of knowledge generation on the topic for several reasons (Arnett & Jensen, 2024; Cozby & Bates, 2020). First, there has not been sufficient prior empirical research on Ubuntu to adequately guide quantitative research; it is not yet clear what questions to best ask, and what language to best use. Second, qualitative interviews are ideal for learning which is not known, allowing participants to frame concepts on their own terms and in their own words—something we considered especially important given the lack of Afrocentric perspectives in mainstream psychology and the lack of empirical research on this topic. Finally, research spanning many decades has demonstrated that interviews are a highly useful measurement approach to understand moral values and development (Jensen, 2020b).

## Method

### Participants

The study included 16 18-year-old Namibians and seven 18- to 20-year-old Kenyans drawn from participants of the Africa Long Life Study (ALLS), a longitudinal study on emerging adulthood in sub-Saharan Africa (Thalmayer et al., 2024). There was an even gender distribution. The sample size was based on the concept of information power, which states that the more information the sample provides, the smaller the sample required (Malterud et al., 2016). A relatively larger sample seemed suitable for the first study in Namibia due to the lack of previous research and the diverse demographics of the sample (described below). For the second study in Kenya, a smaller sample was used because it became clear during data collection that participants largely voiced the same ideas as Namibian participants. To ensure that no new notable concepts were being missed, however, interviews in Kenya were longer to allow for additional follow-up probes.

The Namibian participants resided in three regions of the country. In the capital city of Windhoek (*n* = 5), participants came from “locations” (Otjomuise, Katutura, and Wanaheda) that is, the dense neighborhoods of small concrete homes where Black residents were restricted during apartheid, and where much of the population still lives, and informal settlements (Goreangab), where people live in self-constructed shacks. Near Walvis Bay on the coast, participants came from an informal settlement (Kuisebmund, *n* = 6). In Oshakati region in the northern part of the country, participants were from a rural village (Uukwangula, *n* = 6). The Kenyan participants resided in two sites in Southern Kenya: Nairobi, the capital city (*n* = 4) and Kiserian, a small rural town in Kajiado County (*n* = 3).

These regions represent different ethnolinguistic groups and socioeconomic backgrounds in the two countries, as indicated in Table 1. Although the emerging adults in the two samples varied in many respects, the majority were high school students who lived at home in extended family households and had previously reported in an ALLS quantitative survey either slight or great financial difficulties. This status and living situation are typical of 18- to 20-year-olds in both countries.

**Table 1.** Participant Demographic Information.

Demographic Information	Namibia	Kenya
Gender		
Women	8	3
Men	8	4
Language		
Oshiwambo	12	
Nama/Khoekhoe	2	
Afrikaans	2	
Swahili		4
Gusii		3
Ethnic background		
Oshiwambo	11	
Damara/Nama	3	
Herero	1	
Xhosa	1	
Kisii		5
Akamba		1
Nandi		1
Religious affiliation		
Christianity	15	7
Head of household		
Mother	6	2
Both mother and father	4	3
Extended family	5	
Father	1	
Guardian		2
Employment		
Full-time student	15	7
Part-time work	1	
Relationship status		
Single	12	7
Significant other	4	
Perceived family financial situation		
No problems	6	1
Slight difficulties	7	6
Great difficulties	3	
Household amenities		
Tap water	14	4
Electricity	12	6
Own phone	12	4
Own bedroom	7	2
Functional computer	5	2
Car/truck	1	2
Household size, range	3–15 (*M* = 7.13)	2–8 (*M* = 5.14)

*Note.* The numbers in each column, except household size, represent the number of participants.

### Procedure

Two universities in Namibia and Kenya (University of Namibia, The Catholic University of Eastern Africa) granted IRB approval. All participants provided written consent by signing an informed consent form before participation and received a small airtime voucher (worth 40 NAD and 350 KES). In Namibia, three field-based ALLS research assistants, well known in their communities, assisted in setting up interviews. The individual interviews were conducted by the first author and a Namibian PhD student in August 2022. The first author conducted the interviews in English, and the Namibian PhD student occasionally provided some translation into Oshiwambo. Interviews were audio-recorded with participant consent (*M* = 20.2 min, *SD* = 4.5). In Kenya, three Kenyan ALLS research assistants, including two psychology PhD students and one statistics Master’s student each conducted two or three interviews in English in May 2023 (*M* = 32.3 min, *SD* = 10.3). The first author held a training session and follow-up meetings with the group of interviewers to ensure consistency in the procedure, such as following the structured interview protocol in the same way. In both countries, interviews took place in settings where conversations were unlikely to be overheard, such as a homestead compound, a car, or an empty classroom. All but one participant were either fluent or well-versed in English.

### Materials

For the initial interviews in Namibia, the first author developed the semi-structured interview guide drawing on literature on the topic, in particular on Sodi et al. (2021), in consultation with the last author, the Namibian PhD student, and a master’s student. For the study in Kenya, the first author slightly adapted the interview guide to the Kenyan context in consultation with the Kenyan interviewers. Wording and sentence structures were changed for some questions to retain the same meaning while being easy for participants to understand.

The interview consisted of two parts. The questions in the first part were related to Ubuntu values and behaviors without using the specific term Ubuntu. Questions were instead formulated to reflect associated values and behaviors in simple, natural language. Specifically, there were 14 questions^1^ addressing each of the four elements described in the work by Sodi et al. (2021): African spirituality (e.g., “Do you think that your ancestors have an influence on you and your family now?”), personhood (e.g., “How strongly do you comply with rules and values of your community?”), interconnectedness (e.g., “How important is it for you to be connected to your family and community?”), and communalism (e.g., “Why do you participate in family and community activities?”). There were seven questions exploring possible advantages and disadvantages of Ubuntu values (e.g., “What do you think are positive aspects (benefits) of being part of your community here?” and “Are there any negative aspects (disadvantages) of being part of your community?”). In the second part of the interview, there were four general questions about the terms Ubuntu and harambee themselves (e.g., “Do you know the term Ubuntu?”) to explore participants’ direct associations with the terms. The interview guide is available on the Open Science Framework (https://osf.io/2fcra/).

### Reflexivity and Positionality

The first author is a European woman trained in cultural psychology at European Universities. At the time of the analysis, she had been working as a research assistant for the ALLS for more than 2 years, collaborating closely with Namibian, Kenyan, and South African researchers, and studying African research literature. The Namibian interviewer grew up in Windhoek, with frequent visits to family in the northern Oshiwambo-identified region. She completed a master’s degree in clinical psychology at a Namibian university and is now completing a PhD in Europe. She had prior experience conducting interviews in Namibia. The three Kenyan interviewers grew up in Western Kenya, and now live and work among the Kisii and Kamba tribes included in this study. They are completing MA and PhD studies at universities in Kenya, and all three are experienced interviewers. The second author is an expert in emerging adulthood, globalization, and cultural and developmental psychology. She was closely involved in data analysis, interpretation, and writing. The last author is an expert in cultural and cross-cultural personality psychology and principal investigator of the ALLS. She supported study planning, providing feedback on study design, research question, and interview guide, based on prior experience with qualitative interviews in Namibia and elsewhere.

Throughout the planning, interview, and analysis process, the first author was in continuous contact with Namibian and Kenyan colleagues to develop a culturally informed and emic approach. Additional input from other researchers during data collection and analysis contributed to the rigor necessary for qualitative research (Tracy, 2010). The inclusion or triangulation of the perspectives of several diverse researchers during the interview and analysis process contributes to the validity of findings (Cozby & Bates, 2020). Furthermore, during the conceptualization of the study, data collection, and analysis, the first author kept a self-reflexive journal on how her sociocultural position, subjective lens, and life experiences shaped data collection, analysis, and interpretation of the data. Reflexive journaling allows documentation of impressions and initial thoughts for subsequent interrogation and meaning-making (Braun & Clarke, 2021).

### Analysis

The interviews were first transcribed using SONIX generating a total of 255 pages (149 from Namibian interviews, 106 from Kenya). These were then analyzed with ATLAS.ti using reflexive thematic analysis which can be applied to many different epistemologies and theoretical considerations. It recognizes the researchers’ subjectivity and reflexivity, and values those as integral to the analysis process (Braun & Clarke, 2021). Data analysis followed six steps: (a) Familiarization with the dataset, (b) Coding, (c) Generating initial themes, (d) Developing and reviewing themes, (e) Refining, defining, and naming themes, and (f) Writing up. Specific to this study, this meant identifying emerging adults’ understanding of and identification with values and behaviors related to Ubuntu.

Two rounds of coding were carried out initially, each time with the interviews shuffled in random order. The analysis generated 1,023 codes (722 codes for Namibia and 301 for Kenya), where a *code* is “an analytically interesting idea, concept or meaning associated with particular segments of data” (Braun & Clarke, 2021, p. 53). They were clustered into 24 *subthemes*, where subthemes “share a key concept with a theme of which they are a part” (Braun & Clarke, 2021, p. 85). The subthemes captured the most salient and meaningful aspects of the data. Since reflexive thematic analysis is an iterative process of moving back and forth between the phases, a third round of coding was later completed. The codes and subthemes were synthesized into four emic *themes*. A theme is “a pattern of shared meaning organized around a central concept” and can contain various subthemes (Braun & Clarke, 2021, p. 77). Coding and clustering into subthemes was done separately for each country, with the Namibian interviews being analyzed first, followed by the Kenyan interviews. At the later stages of synthesizing, refining, and defining themes, all interviews were combined because the results indicated that Namibian and Kenyan participants described Ubuntu values in similar ways.

## Results

The four emic themes identified were: (a) I am Because I am Connected, (b) Together We do Better, (c) Following and Passing On Traditions, and (d) Costs of Community: Freeloaders and Restrictions. The first three themes highlight positive aspects and advantages, whereas the fourth pertains to participants’ more negative views of disadvantages to the values and behaviors associated with Ubuntu. Table 2 lists all themes with their associated subthemes. The themes are elaborated below. Quotes are identified by country (N or K), participant number, and gender (w or m).

**Table 2.** Ubuntu Themes and Subthemes.

Themes	Subthemes
I am because I am connected	● A person is defined by her or his connection to the family, community, and environment.
	● The feeling of connection to community and family across generations emerges through shared activities, community participation, mutual support, genes, kindness, sharing, and respect.
	● Being connected to the family and community guarantees support.
	● Connection leads to being on the right path and facilitates teamwork.
	● Connection and support are the key to progress and well-being. The community acts as a safety net.
	● Ancestors positively and negatively influence the current generation and people’s behavior through many mechanisms (e.g., inherited behavior and traits, genes, values, namesake, advice).
	● Emerging adults believe in ancestors and their protection and guidance and feel connected to them.
	● Nature is to be taken care of and protected for well-being and future generations. Emerging adults feel connected to nature.
	● Emerging adults feel connected to and live according to supreme power/God.
Together we do better	● Family and community come first; own needs come second. Preference to help others first.
	● Mutual support (reciprocity) among community and family members is essential.
	● Together we can do better and grow: Preference to do things together as compared to alone (e.g., easier, faster, motivating, leads to connection, success, and better outcomes).
	● Community and family provide support (financial, basic needs, education, motivational, spiritual), guidance, advice on right and wrong, safety and security.
	Education is a means to give back and support the community and family. Expectation to give back after completion of education.
Following and passing on traditions	● Being a community member entails respecting community rules (e.g., to support, share, participate in activities, respect authority and elders).
	● Community expectations imply to support others, pursue education, and give back, follow religious practices, and teach communal values to the younger generations.
	● Not fulfilling expectations implies consequences for the individual (e.g., reduced support and connection).
	● Following community expectations and rules ensures order, safety, and connection and has positive benefits (e.g., success, be role model).
	● Rule compliance is non-negotiable. The opinions of the family and community should be followed.
	● Community expectations are followed first, before individual action.
	● Participation in community activities out of expectation and rule compliance but also out of value to help others, learn from others, and being connected.
	● The community passes on and teaches values (e.g., kindness, respect, sharing, obedience, togetherness), traditions, rules, and behaviors to the next generation.
Costs of community: freeloaders and restrictions	● Following obligations and expectations may mean not following own preferences, decisions, and choices.
	● Helping and supporting others can have disadvantages (e.g., value of helping others can be exploited, people pretend they need help or do not contribute equally, one-sidedness).

### I am Because I am Connected

According to both Namibian and Kenyan emerging adults, following the values and behaviors implied by Ubuntu means that individual identity includes connections with others: “If you are not connected to your environment or your family, you will be a lost soul” (N9w). Two other participants mentioned: “The community and my family is part of me. If I’m connected with them, I feel like I’m still connected with the core part of me” (K7m) and “It is very important to be connected because you need your community” (N6m).

A person’s fundamental connections encompass a broad, interwoven network. They include both family and the local community, which are not strictly differentiated from one another. One person said, “My community is like. . .one family, . . .we work together, we play together” (N8m). Connections are forged through joint activities and decision-making, as another participant explained, “We all cook, fetch water, look after animals, and. . .clean the house” (N4m). Another said, “In everything that my family does, I’m not left out in the process. . .I feel like I’m part of it. I’m connected deep into the roots of it” (K3m). Work and decisions are shared across generations, “The [elderly] surely give space for youth to give something, to add something on a project, or an idea. . . .We are part of it. They make us feel we are inside it” (K3m).

These connections go beyond the presently living to also include ancestors. Participants spoke of ancestral connections in terms of inherited traits: “The meaning of my surname in our language is to be a hardworking person. My [grandfather] had the surname. My uncle has the surname. I have the surname. . .I can see the hardworking trait in us. . .I think it’s the gene in us. . .from my late grandpa [to] my uncle to me to the children now” (K3m). Alongside inherited traits, participants regarded ancestors as active in the community’s everyday life. One stated, “We do believe strongly that the dead are watching over us” (K5w). The presence of ancestors is woven into everyday practices and special events. A participant described how: “Ancestors, they will help you, save you. . . .If there is an accident, the ancestor will come there spiritually [even if] you. . .cannot see it, they save you” (N11w). Another explained, “For instance, [when] you buy a new car, it has to go to the ancestors. They have to look at it, then welcome it, so it doesn’t cause accidents [and] . . .to bless the vehicle from the dangers of the road” (N6m). Connections to ancestors are not only beneficent. One person explained that “In our tribe. . .we believe that there is this ancestral curse. When one did something bad in the community, the ancestors. . .could curse the [person] if they did wrong or made a taboo” (K1w).

Participants described a person’s connections extending even further, into the spiritual world. Worship, prayer, church attendance, participation in church activities (e.g., choir), and adherence to God’s commandments are integral aspects of daily life and passed on to the next generation, providing a spiritual foundation. One participant explained: “We have a routine of waking up as a family together, taking morning prayers and studying the Bible together. Then making breakfast, after that, we usually come together again to pray for our daily activities. . . .Then later we meet in the evening, take dinner, say our prayer, then we sleep” (K3m). Another participant stated: “You have to pray every day as a family, you need to go to church every Saturday or Sunday” (K5w). A third mentioned: “I was raised [learning] how to pray. . . .We go to the church each Sunday, go [to] pray and thank God” (N4m).

Finally, a person is connected to the natural environment. Participants emphasized the vital role of nature in sustaining their well-being and ensuring a future. One participant explained, “My grandma is an herbalist. . . .She usually goes out, takes some herbs, mashes them, and makes something that their neighbors take. And later they come and say that it worked” (K3m). More broadly, another emerging adult explained: “Nature is something we need to all protect because without nature, we all suffer” (N6m).

Thus, the individual sense of self of the emerging adults in our study was braided together with their extended family, their local community, their ancestors who had come before, and the natural environment around them. Whatever aspects of a globalizing, individualistic world they may have experienced, these interconnections were deeply and warmly embraced by the 18- to 20-year-olds we interviewed, in both Namibia and Kenya, in rural and urban contexts, and in more and less affluent situations.

### Together We Do Better

The ways Namibian and Kenyan emerging adults described values and behaviors related to Ubuntu in their lives made vivid the power and benefits of collective effort. Both Namibian and Kenyan emerging adults described how this leads to success and safety, especially in the realm of education.

Every community member and the community as a whole benefit from collective efforts. One participant explained, “I engage [in community activities] because we are one community; if there is a problem, we can solve it together. It’s better to solve it together than solve it on your own” (N8m). Another said, “As a person you need to know that you cannot do work alone. You need some assistance. . . .In life we have to share, we cannot score goals without assistance” (N12m). A third participant elaborated that “several heads are better than one. . . .The time you are in need, people come together, they support you in whatever you want, both spiritually and mentally—and also physically, everything” (K4m). The preference for collective efforts is driven, for example, by the belief that outcomes are achieved easier and work done collectively has greater impact: “Doing things on my own. . .will not contribute as much as being a group because. . .when you are doing something alone, you don’t. . .know whether the outcome is going to be perfect. . .because you need to consult” (K5w). Another motive is mutual dependency: “I will choose to help the other person because. . .you don’t know what may come in the future. I may also need help in future and the same person might be there. If I do not help him, the other time. . .he will tend to not be in a position to help because he did not receive my help when he needed it” (K6m).

Communal collective efforts thereby guarantee success and safety. One participant explained: “I personally. . .cannot make it in this life without the community or my family. With the community and my family, I can succeed” (K1w). Another participant said: “When we help others, we save them from so many challenges that they may go through. And that is a good thing in our community because we will all move forward together” (K6m). A third one mentioned: “It’s all about the community, not an individual. If I want to be successful and succeed in this life, you have to do things as a community. . .do things together as a team. Togetherness and teamwork bring success. When we are together as a community and a family and do something together. . . [we] will do that thing in an easier way, [a] faster way, and a perfect way” (K1w). The community also provides the individual with sense of safety, “when I’m with the community, it’s like you feel safe” (N16m). More concretely, the community provides a safety net. As one participant described, “I’m new in this village, but [the community] supports me. . . .If I need something like money, clothes. . .it’s on their shoulder. They help me a lot” (N4m). The community also assists in difficult situations, such as with hospital visits, or in case of fire and robberies. As one participant said, “As a community, we can stand together and help each other. . . . [When] someone tries to rob maybe a house. . .then we should—as a community—we should help each other” (N5m). The aspiration for collective efforts takes on a distinctive and important meaning for emerging adults. Repeatedly, the emerging adults explained that their families and the community support them in obtaining an education: “Being educated is not coming from my parents. It’s a group of people who came together, sat down, and when my parents went to ask for support, they accepted. And that’s why I’m studying today” (K4m). Support is received “mostly financially such as paying the school fees and even buying food and clothing” (K2w). Another participant elaborated, “In our community when there is someone who is in need of school fees or something. . .they bring up money to take the person to the university” (K1w).

In turn, emerging adults are expected to give back to the community on the completion of their education—to make the community better. One participant explained, “I really need my family. . .when I’m in school and they really need me when I’m done with school” (N5m). Another participant said “in studying I’ll get something which is going to lead me into having a job and I can help [the community] if I have a job. . . .If I’m jobless, how would I be able to help them financially?” (N15m). Moreover, education empowers individuals to bring betterment to the community, as three participants explained: “For studying hard, you can change the condition at home” (N10w), and “If I’m done [studying], I can do something for my family and I can make the community a better place” (N8m), and “They [my family] expect that I’ll pursue an education and have a good job, then change everything at home” (K4m). Some participants had very specific plans for the betterment of their communities, “I want to. . .study hard, get work and get a lot of money and then build something like an orphanage” (N9w) and “I would mostly like to focus more onto my village, provide a better clinic, better hospitals” (N6m). Thus, collective efforts were very important to both Namibian and Kenyan emerging adults, who readily helped others and seemed to enjoy it: “It is usually a very joyous moment, and we are bonding actually” (K6m).

### Following and Passing on Traditions

The third theme pertains to respect for tribal, familial, and community traditions. The emerging adults regarded traditional expectations as obligatory: “Following rules it’s a . . . necessity” (K1w) and “Those are the rules that I must follow” (K2w). One participant explained: “Everything they [community] ask I do. When they say there’s a meeting, I also have to be there, then I will just go. I obey their rules” (N15m).

Adherence to rules was associated with advantages. One participant explained: “I respect [the rules] because when keeping rules, everything is always in order, you must always follow the rules” (N16m). Another participant said, “For one to succeed in this life, you have to follow the rules. Being obedient, you can become successful” (K1w). Another emerging adult said: “[My family] will tell me if I need to change or if I need to continue, if I’m doing the right thing or the wrong thing. I think their opinion is very important and I usually try. . .to please them in my daily activities” (K6m).

Maintaining traditions provides guidance not just in the moment but also across generations. One emerging adult emphasized: “[The community values] teach me. . .more than I know. They teach me how to—when I grow up—how to be a respectful woman and they teach me good values. . . .They give me a lesson into life” (N9w). Another noted that “[My family] teaches us [what] the tradition is like, [and] how we should follow it” (N6m). In turn, the emerging adults anticipated the same expectation of passing on values: “When the younger generation is going to come and I’m going to get old, I also have to teach them [the traditions]” (N15m) and “Elder siblings take up the role of nurturing their younger siblings. . . . I’m also expected to be able to bring up positive traits within my younger brothers and sisters” (K7m). Emerging adults take responsibility for and pass on traditional expectation, “You [will] be a role model to the society. They [will] be like ‘Be like this one. Be like someone that’s good example to the others’” (K5w).

### Costs of Community: Freeloaders and Restrictions

In addition to the positive themes celebrating the power and advantages of Ubuntu, the interviewees brought up two notable disadvantages or costs related to the values and behaviors implied by Ubuntu. One of these, a common concern in any group enterprise, pertains to individuals not contributing their fair share. One participant explained, “You can find someone is lazy in the community. You work as a group, as a team, [and that person] is not part of it. But at the end of the day, you have to share [the outcome of the work] equally” (K5w). Another participant said, “There are some people that will take advantage of being helped. They’ll pretend that they need help [even when] they do not, and that they will just hide whatever they received for themselves” (K4m). Although helping can be abused, not helping is also not an option: “I have no option other than to comply with [community rules] because there’s consequences when you go against them. . . .If you don’t contribute, the [community] will never understand that at that moment you never had something to contribute. . . .They believe that someone who doesn’t give doesn’t belong to that family, to that community. If you don’t give, you are likely to be outcasted from the community because they believe you are an outcast” (K5w).

The other disadvantage noted was about restrictions or lack of freedom, which might conceivably be especially salient to emerging adults. Some chaffed at how their families curtailed their individual educational aspirations, “We have to follow what our parents say. . . .Most of the time. . .academically they choose your courses for you. . .but you’re not passionate about it” (K6m). In similar language, two other participants mentioned: “Sometimes my family and my community, they want me to do something that I’m not interested in” (N7w) and “What they [the community] are going to choose for me is not [what I am] going to be best at” (N15m). Some participants also described their individual religious beliefs being discouraged, “There is no freedom of worship. . . .You have to follow that doctrine so you cannot follow your own religion” (K5w). In short, some of the participants felt constrained by the expectations of their family and community.

### Understandings of the Terms Ubuntu and Harambee

During the second part of the interviews, participants were asked if they were familiar with the specific words Ubuntu and harambee, and what these terms mean to them. The responses to these questions were not included in the thematic analysis but are summarized here.

Most of the Namibian and Kenyan emerging adults were familiar with the term harambee. The Kenyan participants knew the term *Ubuntu*, whereas Namibian participants did not. In Namibia, participants associated harambee with a specific political campaign, governmental support, and the concept of working together. One participant, for example, mentioned the “Harambee prosperity [plan]. . . .Harambee is like talking about presidents. . .ministers” (N11w). Another participant elaborated, “What I understand about [harambee] is that. . .it’s part of the. . .vision 2030. . . .Harambee is. . .a way [to deal] with poverty, whereby the [government] gives free food. . .especially in the village areas. . . . The big truck is there, getting maize, oil, fish cans. . . .They distribute them to the community” (N6m). Indeed, many participants associated harambee with free food: “Harambee is when you’re getting. . .food from the government. . .for free” (N7w) and “[Harambee is] free food and working together” (N14m). The Namibian emerging adults’ understanding of the term harambee, then, was closely linked to the HPP introduced by the Namibian government—a plan aimed at reducing poverty by means of teamwork (Nanyeni, 2016).

In Kenya, the emerging adults associated harambee with togetherness, collaboration, and support. One participant said: “[Harambee means] togetherness. . . .Coming together to accomplish something” (K1w). Similarly, another stated: “[Harambee is] about people coming together, contributing whatever they want. . .to help [those] who are in need in the society” (K4m). One participant explained hearing about harambee through politics: “Our former president, I saw him on television, he was saying Harambee. . .Harambee is. . .uniting us as people, as a community, so that we may work with the same spirit” (K6m). The Kenyan emerging adults were also familiar with the word Ubuntu and associated it with prioritizing the community over the individual. One participant said, “Ubuntu is all about the community, not only an individual person” (K1w). Another mentioned, “Ubuntu is helping others and not individualistically looking at yourself. You look at others. . .and you make sure that they are okay. If you are in a position to help, you help them first. . .before you look at yourself” (K6m). A third participant elaborated a bit more: “The idea of Ubuntu is communalism. It creates teamwork among the society, among the people, and it makes people to share their decision and their problems” (K2w).

## Discussion

Using a cultural-developmental psychology approach, this study aimed to gain an emic understanding of Namibian and Kenyan emerging adults’ views on, identification with, and behaviors shaped by Ubuntu values. Despite calls to integrate and build on African perspectives (Adetula et al., 2022; Nsamenang, 2015), psychological science is still shaped by Western frameworks and samples. This study takes a “Cultural African psychology” orientation, in Ratele’s (2017) influential conceptualization, emphasizing African cultural and spiritual phenomena. Four themes were developed. Three focused on positive, advantageous aspects of Ubuntu values, in terms of identity with the collective, the power of mutual support, and the importance of passing on traditions. One instead highlighted a disadvantage, in terms of freeloaders and restrictions. All four themes encompass aspects relevant across the lifespan, but importantly each also includes aspects distinctive to the current generation of emerging adults.

The analysis began separately for Namibian and Kenyan interviews, but the findings were collapsed when it became clear that their perspectives were highly similar. Although Namibia and Kenya are geographically distant, theoretical work positing pan-African similarities in Ubuntu values were supported (e.g., Adjei, 2019). One possible reason is that most of the participants have an ethnic background and speak a language within the larger Bantu family (e.g., Gusii, Herero, Ovambo, Swahili, Xhosa; “Bantu Languages,” 2024), and values can be transmitted through language (e.g., Mugumbate et al., 2023). The similarities likely also have to do with shared social and contextual experiences in the African region. Prior literature has consistently described Ubuntu values as important across African countries (Adjei, 2019; Mugumbate & Chereni, 2020; Oppenheim, 2012).

### African Personhood, African Community

Similar to their parents, grandparents and other elders, our Namibian and Kenyan emerging adult participants understood full personhood to be communal and to involve responsibility for others. This differs from the focus on individuality and the cultural logic of dignity in Western psychological literature (Adjei, 2019; Leung & Cohen, 2011). Cultures in Africa have often been described using Western definitions of community (e.g., Triandis, 1996). Our results indicate that these definitions do not adequately capture the full spectrum of how community is lived and embraced in Namibia and Kenya, where loyalty to community implies not only close people and adhering to their norms and obligations, as in Western definitions of community (e.g., Markus & Kitayama, 2010; Triandis, 1996). The understanding of community described by our interviewees encompasses a larger sphere, including living in harmony with nature and attending to ancestral and spiritual guidance. This broader understanding of community provides empirical support for the theory of Ubuntu summarized in the work by Sodi and colleagues (2021), among others. Western definitions of community may be useful terms in many cases, but they do not adequately capture the dynamics of African communities.

Thus, the present emic elaboration of Ubuntu enriches our understanding of psychology in Africa and broadens psychological theories and concepts (Serpell et al., 2022). Our results provide opportunities not only to revisit existing psychological theories that have been developed in the West but also to develop new Afrocentric theoretical concepts and research measurements to improve and enrich psychological science and make existing theories more relevant (Adetula et al., 2022; Thalmayer et al., 2021).

This is especially important considering Namibia’s and Kenya’s history of colonialism, where Ubuntu ideals played an important role in decolonization movements (Africa Social Work and Development Network, n.d.). Academic anti-colonialism has fostered the development of indigenous psychology, with the aim to decolonize and indigenize psychology (Hwang, 2005). African psychology, which informs this study, moves beyond the boundaries of Western psychology by including African cultural aspects, such as religiosity, spirituality, ancestors, traditions, and values, among others (Nwoye, 2015; Ratele, 2017).

### Namibian and Kenyan Emerging Adults: A Special Role in Their Communities

The present findings provide empirical support not only for an understanding of Ubuntu that goes back many generations in Africa; the findings also highlight that Namibian and Kenyan emerging adults have a special role in their communities by virtue of their place in the life course and their place in history. Emerging adults may embrace Ubuntu values and behaviors in a way distinct to this generation in their countries. Their communities have high aspirations for their emerging adults and are investing heavily in their secondary and tertiary education. Generational changes make this possible. Unlike their elders, Namibian emerging adults were “born-free” and have new opportunities for tertiary education and urban migration in an independent nation (Lindeke, 2014; Smith et al., 2020). Kenyan emerging adults, called the “dotcom” generation, also have new educational and employment opportunities (Moore & Smith, 2020).

Being given more communal resources to complete their education during emerging adulthood—rather than focusing exclusively on wage earning—brings with it expectations to give back on the part of the community and the emerging adults themselves. The emerging adults’ feeling of obligation to give back to the community was seen when they spoke of adhering to communal expectations and rules but also by their drive and will to change the conditions in their communities. Some interviewees desired to provide better medical clinics, hospitals, or orphanages, and to improve conditions at their homes and towns. These emerging adults hope to bring about changes in their own communities and by extension in society more broadly (see also Lindeke, 2014; Smith et al., 2020). As only a few participants were specific about how they hope to give back to the community, future research could explore the specific aspirations and perceived needs that motivate today’s emerging adults.

The emerging adults in our study live according to values related to Ubuntu philosophy and are eager to teach them to the younger generation, aspiring to become role models in their community in ways that reflect these values. Other studies from around Africa, even those that do not explicitly focus on Ubuntu values, identify similar priorities. For example, South African “born-frees” ages 17–30 years reported being eager to participate in community activities and projects, mentor others, and give back to their communities (Willmore et al., 2023). Cameroonian emerging adults defined becoming an adult as being less self-oriented, developing greater consideration for others, supporting family and significant others financially, finishing school, and contributing to the enhancement of their communities (Lo-oh, 2016).

Despite growing up in a world with more access to technology, education, and political independence, today’s Namibian and Kenyan emerging adults clearly embrace African communalism. They take advantage of education and technology with an eye toward showing respect, giving back, staying connected, and becoming leaders in their community. Our findings show that these emerging adults strive for personal autonomy and achievements to help and take responsibility for others, such as obtaining a job or completing an education to help the family and community. This could be interpreted as a combination of agentic motives (e.g., Little et al., 2006) with communal ones, which may define the core cultural logic of sub-Saharan Africa.

### Emerging Adulthood Today: A Desire for Individual Choices in Education and Religion

As much as the present emerging adults identify with traditional communal values, some of them also departed from the values of their elders. They expressed a desire for individual choice. Unlike previous generations, today’s emerging adults have more opportunities, allowing them to explore diverse paths and individual ambitions. Consequently, their dreams and aspirations may diverge from the expectations set by their families and communities, who may have less knowledge of the possibilities available of all the options to young adults today. As a result, emerging adults may find their individual choices curtailed when adhering to the expectations imposed on them by older generations.

Globalization and education often increase aspirations for individual choices (Jensen, 2022), which may diverge from traditional African values. Media further expands emerging adults’ horizons, granting them a broader perspective on possible goals, and has grown in the last decades around the world: Worldwide internet use has increased from around 1 million users in 2005 to more than 5 billion users in 2024 (Statista, 2024)—many of them African younger people. Among the Kenyan “dotcom” generation, access to new technologies, digitalization, and internet penetration rate contribute to desires for more education, independence from their parents, and autonomous decision-making (Moore & Smith, 2020).

The two areas where the present emerging adults desired more individual autonomy were education and religion. Exploring educational options and attaining education are important goals for emerging adults across contexts (Arnett, 2000, 2016). Education rates are steadily increasing: Primary education completion rates increased from 47% in 1970 (Kenya) and 79% in 1992 (Namibia) to 100% in 2014 and 2018, respectively (The World Bank, 2022). Secondary education completion rates increased from 13% in 1971 (Kenya) and 47% in 1992 (Namibia) to 82% in 2016 and 97% in 2022, respectively (The World Bank, 2024a, 2024b). Research has shown that higher education leads to more autonomy values (Ding & Yu, 2022), which could be a reason for the frustration about curtailment of individual choices expressed by the present emerging adults in cases when elders decide on their offsprings’ school curriculum, courses, or religious beliefs.

Today’s emerging adults also seem to place importance on exploring religiosity and spirituality and deciding on their own beliefs—whether religious, spiritual, or secular (Barry & Abo-Zena, 2014). Our findings might signal a shift in religious affiliations across generations in Namibia and Kenya, where new churches and Christian denominations arise (e.g., Mwila, 2022). Of course, friction between the wishes of emerging adults and their parents regarding education, career, and religious beliefs and practices is seen in many contexts (e.g., Fouad et al., 2008; Mitra & Arnett, 2021; Park & Ecklund, 2007). We also do not know if the parents and grandparents of our interviewees might have felt their own stirrings toward more individual choices when they were 19 years of age.

### Limitations and Future Directions

A limitation of this study is the relatively young age of the emerging adult participants. Falling within the lower end of the emerging adulthood age spectrum of 18–29 years (Arnett, 2000, 2024), their perspectives may not fully reflect the views of emerging adults of the whole age range and the development of views over time. A longitudinal approach could address how views of and identification with Ubuntu values change with age. For example, do our participants maintain their alignment with communal traditions or might they express more reservations about limitations on individual autonomy as they mature? Second, this study used investigator triangulation by including different interviewers and researchers. Future research studies using other forms of triangulation could further enrich insights into Ubuntu (Carter et al., 2014). Such studies might employ method triangulation by combining interviews with structured and ethnographic observations, theory triangulation using different theories for data analysis and interpretation, and data source triangulation by collecting data from different sources, such as individuals, families, or community groups (Carter et al., 2014).

This in-depth qualitative examination of Ubuntu in Namibia and Kenya provides important groundwork for an exciting long-term, mixed-methods research agenda. Future research could build on our findings to develop a questionnaire to measure Ubuntu with larger samples. A subsequent step could involve cross-cultural comparisons of Ubuntu to test its convergent and divergent validity with related, Western-derived cultural concepts, such as individualism/collectivism (Hofstede et al., 2010), power distance (as part of the GLOBE project; House et al., 2002), and tightness–looseness (Gelfand et al., 2006), among others. This approach would enable researchers to assess the differential validity of Ubuntu.

Given that Ubuntu is regarded as a pan-African value, it would be valuable to extend this research program to other African countries with diverse ecological, historical, and sociocultural contexts. This would help determine the ways in which the patterns and values identified here can be considered pan-African, and where differences might lie. Exploring for cultural variation in expressions and correlates of Ubuntu values across African contexts, and beyond, would be in some ways similar to the approach of Kitayama and colleagues (2022) to more systematically define nuances in types of interdependent communities. Ultimately, such an effort could aid the identification of a potentially African-specific cultural logic.

## Conclusion

Living by the values and behaviors implied by Ubuntu and being immersed in the community comes with many benefits and a few negative aspects for Namibian and Kenyan emerging adults. Although our results indicated a desire for more autonomy in the important areas of education and spirituality, it is also clear that the present emerging adults on the whole identified strongly with Ubuntu values and live them in old and new ways in today’s changing world. Our study makes a rare empirical contribution on Ubuntu values among emerging adults, and it contributes insights from an emic, Afrocentric perspective on a philosophy that long has been and continues to be of great importance in this region.

## Notes

1. In the Kenyan interview guide, a few questions were revised to retain the same meaning as in Namibia and to allow for more in-depth questioning after initial analyses in Namibia. For example, there were 13 instead of 14 questions based on the work by Sodi et al. (2021) in the first part, and 3 instead of 4 general questions about the terms in the second part. The order of questions in the first part was also changed slightly.
